# *Leuconostoc* *mesenteroides* Strains Isolated from Carrots Show Probiotic Features

**DOI:** 10.3390/microorganisms9112290

**Published:** 2021-11-04

**Authors:** Emily Schifano, Alberta Tomassini, Adele Preziosi, Jorge Montes, Walter Aureli, Patrizia Mancini, Alfredo Miccheli, Daniela Uccelletti

**Affiliations:** 1 Department of Biology and Biotechnology “C. Darwin”, Sapienza University of Rome, Piazzale Aldo Moro 5, 00185 Rome, Italy; emily.schifano@uniroma1.it (E.S.); adele.preziosi@uniroma1.it (A.P.); jmontes1677@gmail.com (J.M.); 2 Department of Physiology and Pharmacology “Vittorio Erspamer”, Sapienza University of Rome, 00185 Rome, Italy; alberta.tomassini@uniroma1.it; 3 NMR-Based Metabolomics Laboratory (NMLab), Sapienza University of Rome, Piazzale Aldo Moro 5, 00185 Rome, Italy; alfredo.miccheli@uniroma1.it; 4 R&D, Aureli Mario S.S. Agricola, Via Mario Aureli 7, 67050 Ortucchio, Italy; produzione@aurelimario.com; 5 Department of Experimental Medicine, University of Rome, Viale Regina Elena 324, 00161 Rome, Italy; patrizia.mancini@uniroma1.it; 6 Department of Environmental Biology, Sapienza University of Rome, Piazzale Aldo Moro 5, 00185 Rome, Italy

**Keywords:** probiotic, carrots, *Caenorhabditis* *elegans*, pathogen resistance

## Abstract

Lactic acid bacteria (LAB) share several beneficial effects on human organisms, such as bioactive metabolites’ release, pathogens’ competition and immune stimulation. This study aimed at determining the probiotic potential of autochthonous lactic acid bacteria isolated from carrots. In particular, the work reported the characterization at the species level of four LAB strains deriving from carrots harvested in Fucino highland, Abruzzo (Italy). Ribosomal 16S DNA analysis allowed identification of three strains belonging to *Leuconostoc* *mesenteroides* and a *Weissella* *soli* strain. In vitro and in vivo assays were performed to investigate the probiotic potential of the different isolates. Among them, *L.* *mesenteroides* C2 and *L.* *mesenteroides* C7 showed high survival percentages under in vitro simulated gastro-intestinal conditions, antibiotic susceptibly and the ability to inhibit in vitro growth against *Salmonella* *enterica* serovar *Typhimurium*, *Listeria* *monocytogenes*, *Pseudomonas* *aeruginosa* and *Staphylococcus* *aureus* pathogens. In parallel, the simple model *Caenorhabditis* *elegans* was used for in vivo screenings. *L.* *mesenteroides* C2 and *L.* *mesenteroides* C7 strains significantly induced pro-longevity effects, protection from pathogens’ infection and innate immunity stimulation. Overall, these results showed that some autochthonous LAB from vegetables such as carrots have functional features to be considered as novel probiotic candidates.

## 1. Introduction

The Food and Agriculture Organization of the United Nations (FAO) and the World Health Organization (WHO) defined probiotics as live microorganisms that, when administered in adequate amounts, confer a health benefit on the host [1]. According to this statement, probiotics must be safe, and not exert pathogenic effects or show antibiotic resistance genes that could be transferred. Moreover, probiotic strains should be resistant to gastrointestinal conditions, such as stomach acid pH and bile acids, produce antimicrobial compounds and compete with pathogens by stimulating immunity [2]. Furthermore, probiotics’ efficacy should be confirmed in human studies.

Among the various microbial species associated with food, some of them may share probiotic features. The main source of probiotics used in humans is represented by dairy foods, but increasing evidence has highlighted the importance to select probiotics from other sources, such as fresh fruits and vegetables [3]. Indeed, the availability of commercial milk-based products limits their consumption by people who are intolerant or allergic to lactose. Therefore, fruits and vegetables offer healthy alternatives thanks to their large distribution and nutritive value. Among them, carrot (*Daucus carota* L.), as well as being rich in minerals and antioxidants, is reported to be a reservoir of carotenoids, vitamins and fiber [4,5,6]. Many studies on carrots have focused on cultivation, breeding, tissue culture, nutrient content and carotenoid synthesis regulation, while few works deal with microbial composition in terms of potential probiotic bacteria [7,8]. Indeed, the most common probiotics, isolated from fruits and vegetables, include different strains belonging to the lactic acid bacteria (LAB) group. This heterogeneous group of Gram-positive and non-spore-forming bacteria are normally present in food products, involved in numerous fermentation processes and some of them are widely used in industrial processes [9]. The major representatives of this microorganism group are *Lactobacillus*, *Streptococcus*, *Leuconostoc*, *Pediococcus*, *Propionibacterium*, *Enterococcus*, *Bifidobacterium* and *Weissella* genera [10]. Although the *Weissella genus* is found in multiple habitats, many species were isolated from different foods, such as fermented crop products, meat and fish, along with *Leuconostoc* species. Moreover, many of them produce exopolysaccharides, influencing the adhesion to substrates and affecting the structure of fermented foods.

Since the direct evaluation of probiotic potentials in vivo is often expensive and timewasting, the use of simple and inexpensive model systems is needed. *Caenorhabditis elegans* is a powerful in vivo model to screen for probiotic bacteria. Nematodes feed only on microorganisms, which reach the intestine, influencing nematodes’ physiology [11]. Among its many advantages, the possibility to easily monitor anti-aging markers or innate immunity pathways could be used for the screening of microorganisms to identify new probiotic strains and to explore the possible molecular pathways involved. Indeed, several foodborne LAB were reported to exert positive effects in worms, and the mechanisms correlated with innate immunity and lifespan extension have been elucidated [12]. Recently, different *Lactobacillus* strains, isolated from vegetables or dairy products, were reported to increase nematode viability, delay the aging process and protect against foodborne *S. enterica* serovar typhimurium LT2 or *L. monocytogenes* OH pathogens [13,14]. Moreover, it has been demonstrated that *Bifidobacterium* isolates can also exert beneficial effects on *C. elegans* health and lifespan [15].

This study aimed at determining the probiotic potential of four lactic acid bacteria strains isolated from carrots. Tolerance to gastrointestinal conditions, antibiotic susceptibility and antagonism toward human pathogenic microorganisms were evaluated in vitro. The different isolates were tested in vivo using the *C. elegans* animal model to analyze possible beneficial effects on worm lifespan, gut colonization, the aging process and pathogen resistance.

## 2. Materials and Methods

### 2.1. Species Isolation and Identification

Carrots were provided by Aureli Mario S.S. Agricola (Ortucchio, AQ, Italy). The carrot cultivar (*Daucus carota* L., Nantese Dordogne, Syngenta seeds) was grown in Fucino highland (Abruzzo, Italy) and harvested at commercial maturity, as indicated by the supplier’s geneticists. Epidermis and shallow flesh of five carrots (about 20 g) were homogenized with mortar under aseptic conditions and diluted in sterile H_2_O_dd_. Dilution aliquots were plated on De Man Rogosa Sharpe (MRS) medium for 24–48 h at 30 °C, anaerobically. After that, morphologically different colonies were streaked on new MRS plates and grown at 30 °C to isolate purified strains. Each strain was then inoculated in MRS broth anaerobically and, after growth, a stock at −80 °C was carried out.

For bacterial identification, DNA was extracted and amplified according to Schifano et al. [16]. The primer pairs F8 (5′-AGAGTTTGATCCTGGCTCAG-3′) and R1492 (5′-GGTTACCTTGTTACGACTT-3′) were used to amplify the 16S rDNA region of LAB isolates. FASTA sequences of the amplified region from each LAB isolate were submitted to GenBank, and the associated accession numbers are reported in the Results Section.

### 2.2. Growth Conditions of Bacterial Isolates

Bacterial strains isolated from carrots and used in this study were *Leuconostoc mesenteroides* C1, *L. mesenteroides* C2, *L. mesenteroides* C7 and *Weissella soli* T4. The LAB strains described in this work were grown in MRS medium at 30 °C under anaerobic conditions. Commercial probiotic strain *L. rhamnosus* GG ATCC^®^ 53103™ (LGG), used as the LAB reference strain, was grown at 37 °C anaerobically. For *C. elegans* experiments, *Escherichia coli* OP50 strain was used as standard food. For in vitro and in vivo resistance to pathogens, *Pseudomonas aeruginosa* ATCC 15692*, Staphylococcus aureus* ATCC 25923, *S. enterica* serovar typhimurium LT2 and *L. monocytogenes* OH were used. *E. coli* OP50 and pathogen strains were grown in Luria-Bertani (LB) broth at 37 °C overnight, under shaking.

### 2.3. Resistance to Lysozyme, Acid pH and Bile Salts

Isolates were grown in MRS broth overnight at 30 °C. For the lysozyme tolerance assay, 10 mL of overnight culture was centrifuged at 5000 rpm at 4 °C and suspended in the same volume of SES buffer (0.22 g/L CaCl_2_, 6.2 g/L NaCl, 2.2 g/L KCl, 1.2 g/L NaHCO_3_) containing 0.1 mg/mL of lysozyme (Sigma-Aldrich, St. Louis, MO, USA) [17]. After 30 min and 2 h of incubation at 37 °C, 100 μL of each suspension was plated on MRS agar plates and further incubated at 37 °C for 24 h, under anaerobic conditions. SES without lysozyme was used as a control.

The acid tolerance assay was performed according to [18], with some modifications. A 1 mL aliquot of overnight culture (10^9^ cfu/mL) was inoculated into 10 mL of sterile phosphate-buffered saline (9 g/L NaCl, 9 g/L Na_2_HPO_4_ 2H_2_O, 1.5 g/L KH_2_PO_4_) adjusted to pH 2.5 and pH 3.0 with 8M HCl. pH 5 buffer was used as a control. The tubes were incubated at 37 °C and the viable organisms were recovered after 3 h of incubation on MRS agar incubated for 48 h at 30 °C. For resistance to bile salts, the same protocol was performed, using phosphate-buffered saline with 0.3% bile salts (Sigma-Aldrich).

The viability was measured as percent viability = [(CFU_treated_/mL)/(CFU_untreated_/mL)] × 100. The untreated value corresponds to plate counts of inoculated bacteria in control phosphate-buffered saline, and the treated value corresponds to the bacterial counts obtained after incubation in simulated GI conditions.

### 2.4. Antibiotic Resistance

For the susceptibility test, antibiotic discs (Biolab Zrt., Budapest, Hungary) were used. The experiment was performed as described in [13]. Briefly, 100 μL of overnight cultures of different isolates or LGG were plated onto MRS agar plates; then, the antibiotic discs were gently placed on the plates and incubated under anaerobic conditions for 24 h at 37 °C. The zones of inhibition were measured from the center of the disc, recorded and compared with those of the reference strain.

### 2.5. Antimicrobial Activity

The agar diffusion test was performed using, as indicator strains, *P. aeruginosa* ATCC 15692,* S. aureus* ATCC 25923, *S. enterica* serovar typhimurium LT2 and *L. monocytogenes* OH. To evaluate the antagonistic activity of LAB isolates against the different pathogens, 100 μL each of LAB overnight cultures was spotted onto MRS agar and coated with 5 mL of TSA soft agar (0.7%), previously inoculated with 500 μL of each pathogen indicator strain. Plates were incubated at 37 °C for 24 h. The antagonist activity was recorded as the diameter (mm) of growth inhibition halo around each spot.

### 2.6. C. elegans Strain and Lifespan Assay

The wild-type *C. elegans* strain, Bristol N2, was grown at 16 °C on Nematode Growth Medium (NGM) plates plated with *E. coli* OP50. Fertile N2 adults were placed to lay embryos for 8 h on peptone-free NGM plates, plated with LAB strains, LGG or *E. coli* OP50, and then sacrificed. For the preparation of the bacterial lawns, overnight cultures were centrifuged for 15 min at 6000 rpm. The pellet was weighed and suspended in M9 buffer in order to obtain a final concentration of 400 mg/mL. Then, 25 µL of each type of bacterial lawn was plated on mNGM, as described in [19]. When the progeny became fertile (t0), 60 worms per condition were transferred to new plates plated with fresh bacterial cultures and monitored daily. A worm was considered dead when it did not respond to touch. 

### 2.7. Fertility Assay

As described in [20], synchronized worms obtained as above were incubated at 16 °C on mNGM plates plated with different strains, allowing embryo laying. Animals were transferred onto new plates every day and the number of progeny was documented until the mother worms became infertile.

### 2.8. Colonization Assay of C. elegans Gut

For each condition, 10 L4 larvae or 8-day-old adults were washed in M9 buffer and lysed, as described in [21]. Whole worm lysates and serial dilutions were plated onto MRS-agar plates. The number of colony-forming units (CFU) was counted after 24 h of incubation at 37 °C, anaerobically. Instead, OP50-fed worm lysates were plated onto LB-agar and incubated at 37 °C.

### 2.9. Aging Markers’ Analysis

For the pharyngeal pumping rate, the number of grinder contractions was counted under a Zeiss Axiovert 25 microscope in 10-day-old adult animals fed different bacteria from embryo hatching. Ten worms were analyzed for each treatment, during a period of 30 s. The locomotion ability of nematodes was analyzed by body bending counting after 30 s. In particular, as described in [14], 10 worms for each treatment were washed in M9 buffer to remove bacteria, and then placed in 10 μL of M9 buffer to facilitate the locomotion measure. For lipofuscin accumulation analysis, 10-day-old adult worms, after washes in M9 buffer, were placed onto a 3% agar pad containing 20 mM of sodium azide. Afterwards, nematodes were observed with the Axio Observer Z1 inverted microscope, equipped with an ApoTome.2 System (Carl Zeiss Inc., Oberkochen, Germany). Digital images were acquired with the AxioCam MRm high-resolution digital camera (Zeiss) and processed with the AxioVision 4.8.2 software (Zeiss). ApoTome optical sectioning images of animals were recorded under a 40 Å~/0.75 objective (Zeiss). Median fluorescence intensity was analyzed using the ImageJ software, measuring the ratio of pixels per area of the worm.

### 2.10. Resistance to Pathogens in C. elegans

For the killing assay, 35 mm NGM plates were seeded with 60 μL of *L. mesenteroides* C2 or *L. mesenteroides* C7 mixed with different pathogens, in a 1:1 ratio. *Pseudomonas aeruginosa* ATCC 15692 and *Staphylococcus aureus* ATCC 25923 were chosen as representative pathogens of Gram-negative and Gram-positive bacteria, respectively. The assay was performed as described in [22]. Synchronous L4 larvae were placed onto the different co-cultures and incubated at 25 °C. Lifespan was monitored daily and worms fed with pathogen alone were taken as the control. A worm was considered dead when it failed to respond to touch.

### 2.11. Real-Time qPCR

At the stage of 1-day-old adults, 200 worms for each condition were lysed and total RNA was extracted as described in [23]. *pmk-1*, *skn-1*, *daf-16*, *sod-3* and *hsf-1* mRNA levels were analyzed. The differences between the mean CT value of each sample and the CT value of the housekeeping gene (*act-1*) were calculated. Primers used in this study are reported in Table 1. The experiment was carried out in triplicate.

### 2.12. Statistical Analysis

All experiments were performed at least in triplicate. Data are presented as mean ± SD. The statistical significance was determined by Student’s *t* test or one-way ANOVA coupled with a Bonferroni post-test (GraphPad Prism 5.0 software, GraphPad Software Inc., La Jolla, CA, USA). Differences with *p*-values < 0.05 were considered significant and were indicated as follows: * *p* < 0.05, ** *p* < 0.01 and *** *p* < 0.001.

## 3. Results

### 3.1. Bacteria Isolation

In this study, potential probiotic properties of four bacterial isolates were evaluated in vitro and in vivo. Different bacterial colonies were isolated from carrots and identified at the molecular level by the amplification of 16S rDNA. The sequences obtained from sequencing were compared with those in the BLAST database, so that three strains belonging to *Leuconostoc mesenteroides* and a *Weissella soli* strain were identified. The Gram-positive strains isolated were identified as *L. mesenteroides* C1 (accession number OK513088), *L. mesenteroides* C2 (accession number OK513089), *L. mesenteroides* C7 (accession number OK513090) and *Weissella soli* T4 (accession number OK513091).

### 3.2. In Vitro Tests

#### 3.2.1. Resistance to Lysozyme, Low pH and Bile Salts

**Figure 1:**
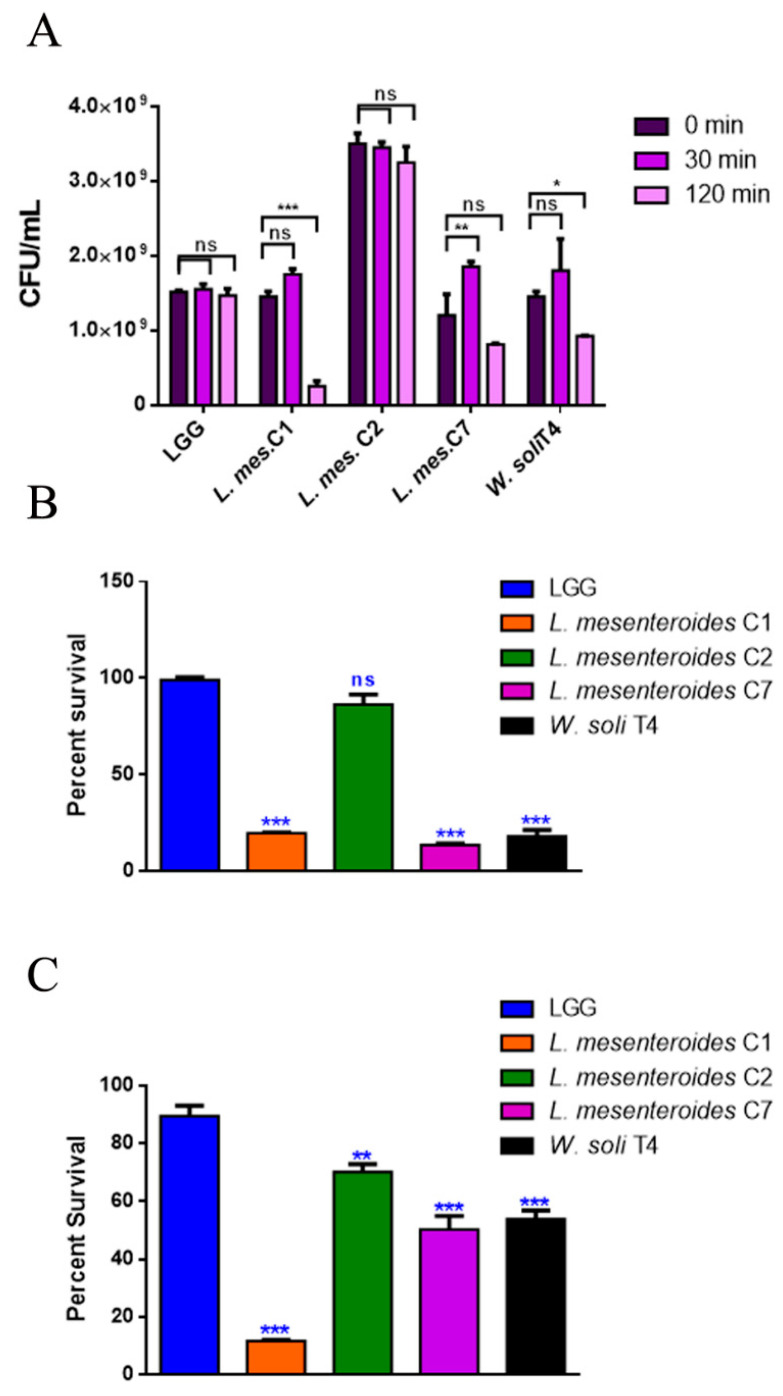
In vitro tolerance to lysozyme, pH 2.5 and 0.3% bile salts. (**A**) Cell counts of viable bacteria recovered at the initial time point (t0), following 30 or 120 min of incubation in 1 mg/mL of lysozyme SES buffer. (**B**) Recovery of viable bacteria after 3 h of incubation in phosphate buffer adjusted to pH 2.5 or (**C**) 0.3% bovine bile salts. LGG was taken as the LAB reference strain. Columns represent the mean ± SD of three independent experiments. Statistical analysis was performed by one-way ANOVA, followed by the Bonferroni post-test. Asterisks indicate significant differences (* *p* < 0.05; ** *p* < 0.01; *** *p* < 0.001), ns: not significant.

To perform a selection of possible probiotic candidates, their resistance to the extreme conditions of the gastrointestinal tract, such as low pH in stomach and bile in the upper intestine, was evaluated. The high concentration of lysozyme present in the mouth represents the first barrier. Figure 1A reports bacteria survival data after 30 and 120 min of treatment with 1 mg/mL of lysozyme. All LAB strains showed high resistance to lysozyme after 30 min of incubation, with percentages of survival of 100%. Notably, *L. mesenteroides* C2 was able to resist even after 120 min of incubation with lysozyme, which can be considered a severe treatment. On the other hand, *W. soli* T4 and *L. mesenteroides* C7 showed a percent survival of 60% and 80% respectively, after 2 h of incubation. *L. mesenteroides* C1, instead, did not resist lysozyme treatment. Tolerance to low pH conditions was performed to simulate microbial flux along the mammalian gastrointestinal tract. As shown in Figure 1B, 3 h of incubation in pH 2.5 exerted a strong reduction (about 80%) of *L. mesenteroides* C1, *L. mesenteroides* C7 and *W. soli* T4 counts, as compared to the control. This result suggested that these strains were not able to endure acidic environments. Notably, *L. mesenteroides* C2 cell recovery showed a higher ability to survive in low pH conditions, similarly to the probiotic LGG strain. Similar results were obtained after testing the ability of different strains to resist at pH 3.0. 

The human bile concentration ranges from 0.3% to 0.5%, and a high bile tolerance improves probiotics’ colonization in the host GI tract [24]. To investigate the ability of the different isolates to survive in the presence of bile, the percent survival in the presence of 0.3% bile was tested. As shown in Figure 1C, *L. mesenteroides* C2, *L. mesenteroides* C7 and *W. soli* T4 strains showed a growth percentage above 50% in the presence of bile. As expected, the reference probiotic strain LGG exhibited a resistance of about 90%. On the other hand, *L. mesenteroides* C1 was not able to resist the treatment, showing a decrease in viability of about 90%.

#### 3.2.2. Antibiotic Susceptibility and Antagonistic Activity to Pathogens

Antibiotic susceptibility was determined by the disc diffusion assay, analyzing a panel of 20 antibiotics. Among them, there are inhibitors of synthesis of cell wall, DNA and RNA, proteins and inhibitors of membrane function. As reported in Table 1, LAB isolates showed an antibiotic susceptibility pattern very similar to that of the LGG control strain. *L. mesenteroides* C2 displayed resistance to only five antibiotics, and in some cases, the inhibition halo was larger as compared to the control (Table 2).

The antagonistic activity was evaluated through the agar double-layer diffusion test, against four pathogen test strains: the Gram-positive *Staphylococcus aureus* and *Listeria monocytogenes*, and the Gram-negative *Pseudomonas aeruginosa* and *S. enterica* serovar typhimurium LT2. The antagonistic activity was less variable among the different isolates, as shown by inhibition halo diameters on all four pathogen test strains (Table 3). Indeed, the inhibition halo diameters produced by the different isolates were comparable to that of the probiotic strain LGG.

### 3.3. In Vivo Tests

#### 3.3.1. Effects on *C. elegans* Lifespan and Colonization Capability

**Figure 2:**
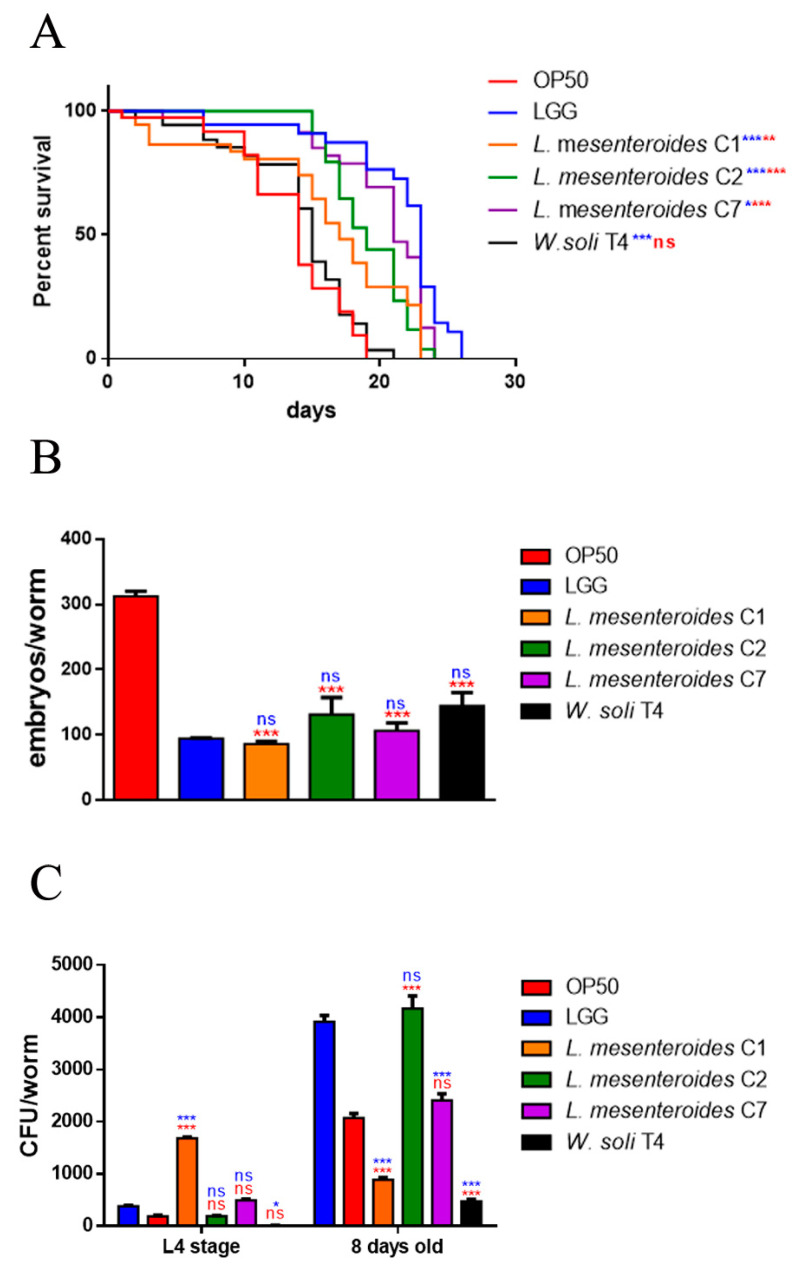
Effects of different isolates on *C. elegans* lifespan. (**A**) Kaplan–Meier survival plot of N2 worms fed *L. mesenteroides* and *W. soli* strains. Lifespans of OP50- and LGG-fed animals are reported as controls; *n* = 60 for each data point of single experiments. (**B**) Average embryos’ production per worm of nematodes fed different bacterial isolates. (**C**) Bacterial colony-forming units (CFU) recovered from L4 larvae and 8-day-old adults fed the four isolates or LGG (LAB reference strain) and OP50 controls. Bars represent the mean of three independent experiments. Asterisks indicate significant differences (* *p* < 0.05, ** *p* < 0.01, *** *p* < 0.001) as compared to LGG (blue asterisks) or OP50 (red asterisks) controls, ns: not significant.

In vivo screening of the four strains was performed in the *C. elegans* model system, to test possible beneficial effects exerted by LAB. For this purpose, worms were separately fed each of the isolated strains starting from embryo hatching, using animals fed LGG or standard *E. coli* OP50 as control populations. Among the tested strains, *L. mesenteroides* C2 and *L. mesenteroides* C7 induced a relevant increase in *C. elegans* viability (Figure 2A), showing similar survival as compared to those fed the probiotic strain LGG. Indeed, 50% of viability was recorded at days 17, 18 and 21 in *L. mesenteroides* C1, *L. mesenteroides* C2 and *L. mesenteroides* C7 fed nematodes respectively, in comparison with day 22 in LGG-fed nematodes. On the other hand, only *W. soli* T4 showed a similar worm lifespan when compared to the control OP50 diet, with 50% of viability recorded at days 14 and 12, respectively. To test possible effects on fertility, progeny production was evaluated. Like probiotic LGG, the brood size of worms fed different isolates showed a reduction of about 60% of the progeny number compared to the OP50 control (Figure 2B). Afterwards, the gut colonization capability was explored by plating worm lysates at different time points and by CFU counting. Results highlighted the increase of all bacterial strains along the lifespan (Figure 2C). At the L4 stage, the CFU number relative to *L. mesenteroides* C1 resulted to be about 2-fold higher than that relative to controls. At the stage of 8 days of adulthood, instead, the gut colonization capability of *L. mesenteroides* C1 was about 80% lower than that of probiotic LGG. On the other hand, in adult worms, *L. mesenteroides* C2 resulted to be able to colonize *C. elegans* gut, similarly to the LGG control. Along worms’ lifespan, *L. mesenteroides* C7 and *W. soli* T4 showed a colonization capability similar to the OP50 strain.

#### 3.3.2. Effects on *C. elegans* Aging Processes

**Figure 3:**
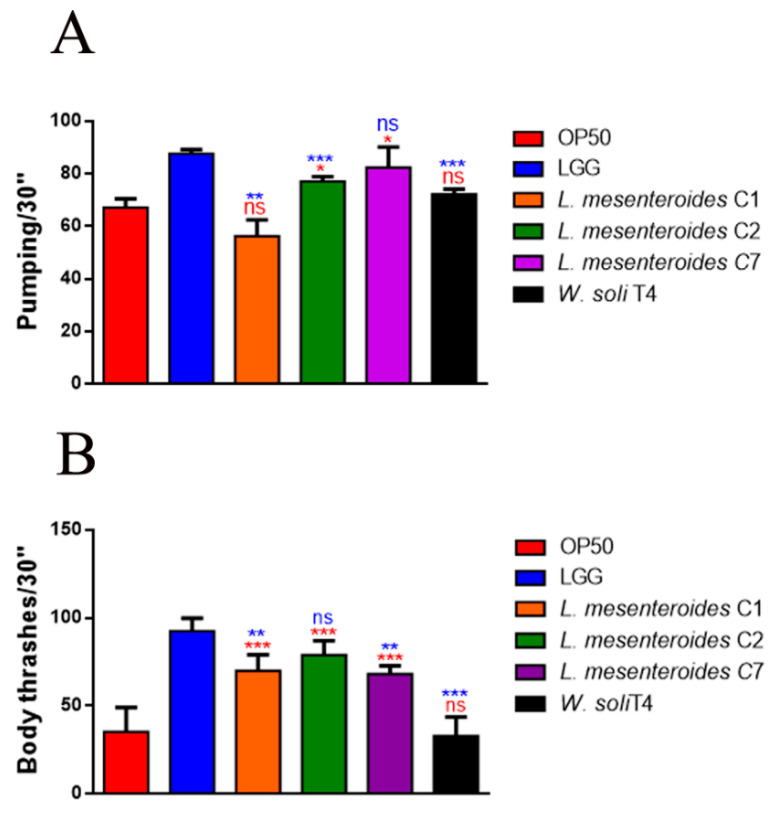
Impact of the different isolates on *C. elegans* aging markers. (**A**) Pumping rate of 10-day-old worms measured for 30 s. 10 worms were used for each condition. Worms fed OP50 or LGG were taken as controls. (**B**) Body bending of *C. elegans*-fed isolates as compared to LAB reference strain LGG or OP50, measured for 30 s. Bars represent the mean of three independent experiments. Statistical analysis was evaluated by one-way ANOVA with the Bonferroni post-test. Asterisks indicate significant differences (* *p* < 0.05, ** *p* < 0.01, *** *p* < 0.001) as compared to LGG (blue asterisks) or OP50 (red asterisks) controls, ns: not significant.

**Figure 4:**
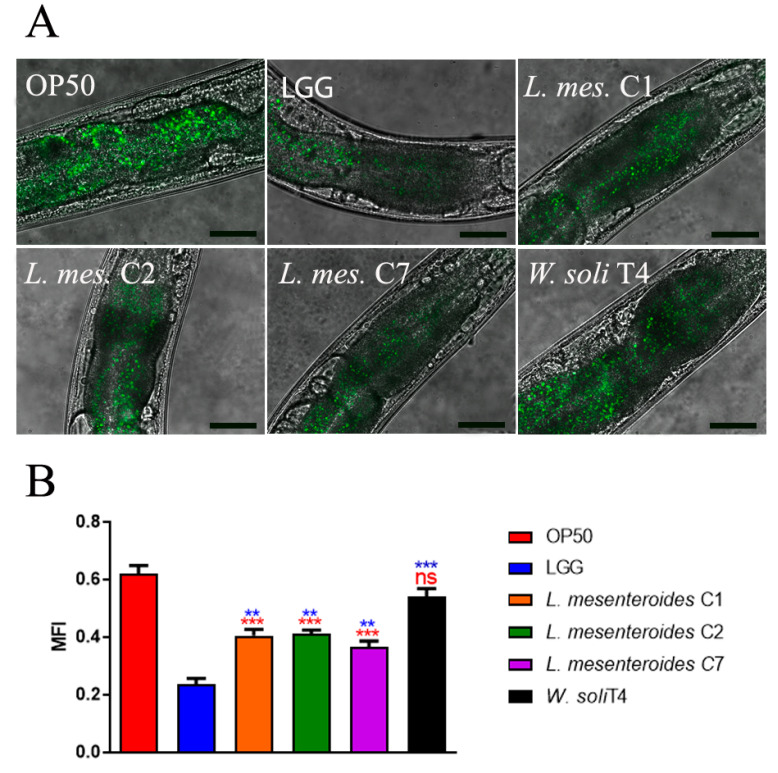
Evaluation of lipofuscin accumulation in *C. elegans*. (**A**) Autofluorescence of lipofuscin granules in *C. elegans* fed different LAB on day 10. Ten worms were used for each measurement. LGG- (LAB reference strain) and OP50-fed worms were used as controls. Scale bar = 50 μm. (**B**) Median fluorescence intensity of nematodes’ lipofuscin. Statistical analysis was evaluated by one-way ANOVA with the Bonferroni post-test. Asterisks indicate significant differences (** *p* < 0.01, *** *p* < 0.001) as compared to LGG (blue asterisks) or OP50 (red asterisks) controls, ns: not significant. Bars represent the mean of three independent experiments.

To investigate whether the pro-longevity effects exerted by LAB strains positively correlated to a delay in aging, age-related biomarkers, such as pumping, locomotion and lipofuscin accumulation, were analyzed. The pharyngeal pumping rate measures grinder contractions associated with food intake ability, normally declining with age. Figure 3A showed that *L. mesenteroides* C7-fed worms share a significantly high pumping rate, at 10 days of adulthood, similarly to LGG-fed worms. Moreover, nematodes fed with *L. mesenteroides* C2 and *W. soli* T4 showed an increase in grinder contraction of 12% and 8%, as compared to OP50-fed worms, respectively (Figure 3A). On the other hand, in *L. mesenteroides* C1-fed worms, a reduction in pumping rate of about 40% with respect to LGG was observed. Then, the locomotion rate of *C. elegans* was evaluated at day 10 of adulthood. In this case, nematodes fed different *L. mesenteroides* strains displayed a higher motility than OP50-fed worms, while locomotion of *W. soli* T4-fed worms was similar to the control (Figure 3B). Furthermore, accumulation of auto-fluorescent lipofuscin is also an aging marker of cellular impairment. Indeed, 10-day-old adult nematodes, when fed *L. mesenteroides* isolates, showed a reduced fluorescence compared to OP50-fed adults, while *W. soli* T4-fed animals showed a higher accumulation of fluorescent granules along the intestine, usual in old animals (Figure 4A,B).

#### 3.3.3. Pathogen Resistance and Innate Immunity Stimulation

Among the four LAB isolates, *L. mesenteroides* C2 and *L. mesenteroides* C7 strains resulted as the most promising candidates in terms of beneficial features. Since a good probiotic is reported to compete with pathogens, protecting the host from infections, the *C. elegans* killing assay was performed to test possible protection from infection by the two isolates. *S. aureus* and *P. aeruginosa* were chosen as representatives of the Gram-positive and Gram-negative group, respectively. As shown in Table 4, nematodes displayed reduced survival when fed pathogens alone, as compared to nematodes fed co-cultures of the same pathogen with *L. mesenteroides* C2 and *L. mesenteroides* C7. Interestingly, 50% of viability was recorded at day 6 in nematodes fed co-cultures of *L. mesenteroides* strain *P. aeruginosa*, in comparison with day 3 of nematodes fed the pathogen alone. Similarly, *L. mesenteroides* C2 and *L. mesenteroides* C7 were able to protect worms from *S. aureus* infection, with 50% of viability recorded at day 7, with respect to day 5 in worms fed *S. aureus* alone.

**Figure 5:**
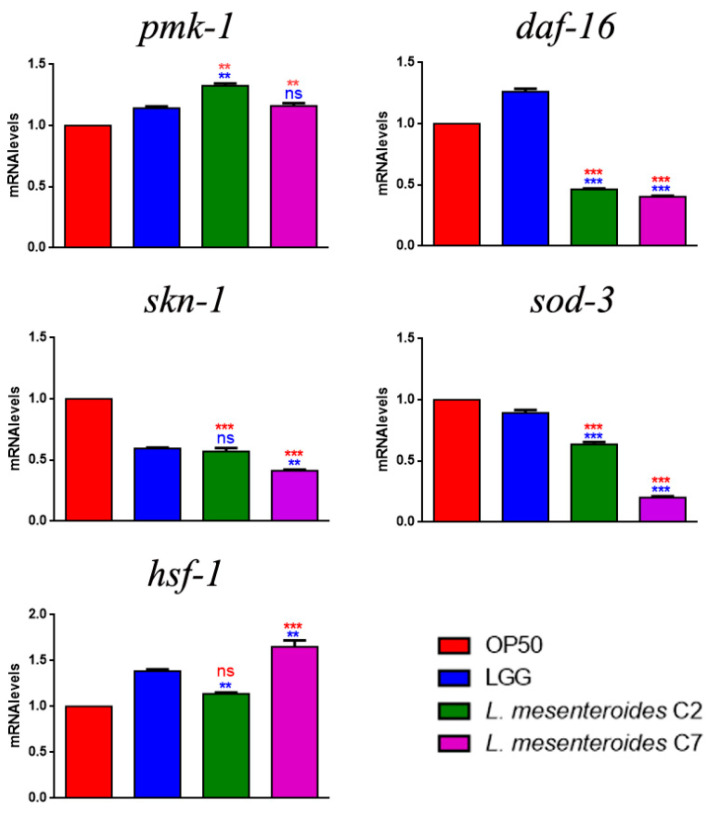
Transcript levels of genes involved in *C. elegans* immunity. Expression of *pmk-1*, *skn-1*, *sod-3*, *daf-16* and *hsf-1* genes in 1-day-old adults fed with different bacterial strains. Experiments were performed in triplicate. LGG (LAB reference strain) and OP50 were used as controls. Data are presented as mean ± SD. Asterisks indicate significant differences (** *p* < 0.01, *** *p* < 0.001) as compared to LGG (blue asterisks) or OP50 (red asterisks) controls; ns: not significant.

To study whether *L. mesenteroides* C2 and C7 could stimulate nematodes’ innate immunity, transcript levels were analyzed for *pmk-1*, *skn-1*, *sod-3*, *daf-16* and *hsf-1* genes, whose activation after probiotic feeding have been described [15,25]. Interestingly, in *L. mesenteroides* C2- and C7-fed nematodes, significantly increased expressions of *pmk-*1 and *hsf-1* transcripts were observed, similarly to LGG (Figure 5). On the other hand, while in LGG-fed worms DAF-16 also seemed to be activated, the two isolates were not able to stimulate this pathway. The expression of genes involved in detoxification processes (*skn-1* and *sod-3*) were instead very low, as compared to the OP50 control, but similar to LGG.

## 4. Discussion

The human microbiota represents the first defense barrier against gut colonization by pathogens. This defense is due to microbiota competition with pathogenic bacteria, preventing their adhesion and subsequent internalization. Indeed, some commensal strains share adhesion receptors with higher affinity than pathogens, also competing for the availability of nutrients and trophic substances. Another strategy adopted by microbiota species is the production of antimicrobial substances called bacteriocins. Therefore, probiotics commonly share the ability to compete for receptor sites, nutrients and trophic substances, and they are able to synthesize bacteriocins. Recently, growing attention in pro-longevity effects exerted by different LAB strains allowed the development of several probiotic products [26]. In particular, fermented foods are the main source of LAB [14,19,27,28], but fruits have also been cited several times for the isolation of interesting LAB [29,30,31]. Due to the probiotics’ relevance to human health, it is pivotal to characterize bacterial isolates to be used in alternative food products. In this context, the main objective of this work was the isolation of LAB from carrots and the selection of strains with potential to be used as probiotic microorganisms. The root carrot (*Daucus carota* L.) is one of the most important vegetables cultivated and consumed worldwide, rich in bioactive compounds, such as provitamin A [5,7]. It is also rich in dietary fiber, antioxidants and other nutrients, but especially in carotenoids. In this work, a combination of in vitro and in vivo methods was used to screen for new potential probiotic *Leuconostoc* and *Weissella* strains deriving from carrots grown in Fucino highland (Abruzzo, Italy). Characterization of LAB isolates at the species level identified three strains belonging to *Leuconostoc mesenteroides* and a *Weissella soli* strain. Indeed, a large percentage of probiotic microorganisms belongs to the LAB group. *Leuconostoc* and *Weissella*, together with *Lactobacillus* and *Pediococcus,* are important genera of LAB associated with foods and fermented products, such as meat, vegetables, dairy and bakery products, and also act as flavoring and texturizing agents [32]. This work showed that, among all tested strains, *L. mesenteroides* C2 and *L. mesenteroides* C7 could survive against the stress conditions assayed in this study. Survival to the adverse environment of the stomach is a key pre-requisite for effective colonization by a probiotic strain [33]. At first, tolerance to gastrointestinal conditions was carried out by evaluating the viability of each isolate in comparison with the commercial reference strain *L. rhamnosus* GG. Notably, *L. mesenteroides* C2 and *L. mesenteroides* C7 displayed survival rates equal to or higher than that of the commercial probiotic control LGG after the different treatments. Only in the case of long exposure to lysozyme and low pH resistance did *L. mesenteroides* C7 show a reduced recovery of viable cells. If this strain will result as positive in subsequent tests, this problem could be solved by encapsulating the bacteria cells, making them viable in the human gut. Furthermore, antibiotic resistance profiling of all isolates resulted similar to probiotic LGG, and this is an important trait to be verified for safety purposes [34]. In this study, different groups of antibiotics were used: cell wall inhibitors, inhibitors of protein synthesis and inhibitors of DNA and RNA synthesis. A recurrent ingestion of these types of antibiotics may cause imbalance in the intestinal sensitive microbiota. Moreover, the antibiotic resistance in probiotics usually does not constitute a safety issue, since resistance genes could be transferred to potential pathogens. It has been demonstrated that probiotics are able to prevent infections by foodborne pathogens, through different mechanisms, such as competitive exclusion or antimicrobial molecules’ production [35,36]. Indeed, LAB show various antimicrobial features, such as the production of organic acids, cyclic dipeptides, phenylacetic acid, hydrogen peroxide, low molecular weight compounds, protein compounds, bacteriocins and fatty acids [37,38,39,40]. We therefore tested the antimicrobial activity exerted by *L. mesenteroides* C2 and *L. mesenteroides* C7 strains against common pathogens, such as *Pseudomonas aeruginosa* and *Staphylococcus aureus*. The isolates resulted to counteract the pathogens in vitro and in vivo. In parallel, the isolated strains were also analyzed evaluating possible beneficial properties in the in vivo model of *C. elegans*. Nematodes commonly feed on bacteria, but a significant number of bacterial cells escape the grinder contractions and can proceed to colonize the worm gut [41]. The *L. mesenteroides* C2 and C7 strains were able to significantly increase *C. elegans* lifespan as compared to the OP50 control strain, similar to the effect exerted by the reference strain LGG. The impact of *L. mesenteroides* C2 and C7 on *C. elegans* physiology could be due to the high gut colonization capacity of the bacterial strains. Moreover, the pro-longevity effects observed in lifespan experiments were associated with the anti-aging effects, highlighted by analyzing different aging markers, such as pharyngeal pumping rate, brood size and lipofuscin. These data further demonstrate the ability of specific LAB strains to prolong nematodes’ lifespan, as described in previous studies [41,42,43,44]. As discussed above, the different isolates also displayed health-promoting activities in host defense against Gram-positive and Gram-negative pathogens in vivo, increasing the survival of infected worms. In *C. elegans,* host–pathogen interactions have been studied for a number of pathogens of human and animal origin [45], including *P. aeruginosa* and *S. aureus,* which colonize the worm gut and infect the nematode [46]. Moreover, innate immunity responses and lifespan were strongly correlated in nematodes [15]. Indeed, worms do not have adaptative immunity, but only innate immune defenses that have many aspects similar to human pathways [47]. Among them, the p38 MAPK and IIS pathways, the transforming growth factor-beta (TGF-beta) and the beta-catenin signaling pathways, are more conserved in humans and nematodes and they can be induced by probiotics [48]. In agreement with these works, real-time analysis highlighted the activation of *pmk-1* and *hsf-1* pathways, suggesting a stimulation of *C. elegans* immunity, which correlates with a reduction of oxidative stress, leading to the pro-longevity and anti-aging effects.

## 5. Conclusions

Among the different strains isolated from carrots, *L. mesenteroides* C2 and *L. mesenteroides C7* showed interesting probiotic characteristics, such as greater lysozyme, pH and bile tolerance, in vitro suppression of pathogen growth and in vivo beneficial effects, exerted on the *C. elegans* animal model. However, in vivo analysis on animal or human systems should be performed to further test their potential beneficial properties for human health.

## Figures and Tables

**Table 1:** Primers for real-time qPCR analysis.

*hsf-1*	FOR	5′-ATGACTCCACTGTCCCAAGG
	REV	5′-TCTTGCCGATTGCTTTCTCT
*pmk-1*	FOR	5′-AAATGACTCGCCGTGATTTC
	REV	5′-CATCGTGATAAGCAGCCAGA
*sod-3*	FOR	5′-AGAACCTTCAAAGGAGCTGATG
	REV	5′-CCGCAATAGTGATGTCAGAAAG
*act-1*	FOR	5′-GAGCGTGGTTACTCTTTCA
	REV	5′-CAGAGCTTCTCCTTGATGTC
*skn-1*	FOR	5′-GTTCCCAACATCCAACTACG
	REV	5′-TGGAGTCTGACCAGTGGATT
*daf-16*	FOR	5′-TCAAGACCTCAAAGCCAATCAACTC
	REV	5′-ACGAGAAAGAAGGAGTAAGAGGAGG

**Table 2:** Resistance to antibiotics of different isolates. The zones of inhibition were measured from the center of the disc and recorded in mm ± SD. Absence of inhibition halo was indicated as (+), ns: not significant.

Antibiotic	Amount on Disc (µg)	Zone of Inhibition (mm)					
		LGG	*L. mesenteroides* C1	*L. mesenteroides* C2	*L. mesenteroides* C7	*W. soli* T4	*p*-Value
Amikacin	30	4 ± 0.03	5 ± 0.06	10 ± 0.08	4 ± 0.05	4 ± 0.03	*p* < 0.001
Ampicillin	10	7 ± 0.06	7 ± 0.05	6 ± 0.10	5 ± 0.20	5 ± 0.05	*p* < 0.01
Aztreonam	30	+	+	+	+	+	ns
Carbenicillin	100	8 ± 0.08	7 ± 0.03	5 ± 0.09	7 ± 0.03	5 ± 0.08	*p* < 0.01
Cefalotin	30	+	3 ± 0.10	+	4 ± 0.08	+	*p* < 0.01
Cefotaxime	30	7 ± 0.08	+	7 ± 0.20	+	+	*p* < 0.001
Cefuroxime	30	5 ± 0.02	+	12 ± 0.08	+	+	*p* < 0.001
Clindamycin	2	8 ± 0.12	9 ± 0.35	4 ± 0.08	10 ± 0.10	9 ± 0.30	*p* < 0.01
Chloramphenicol	30	8 ± 0.08	8 ± 0.35	14 ± 0.10	8 ± 0.20	10 ± 0.15	*p* < 0.01
Erythromycin	15	8 ± 0.03	7 ± 0.08	8 ± 0.15	7 ± 0.09	8 ± 0.08	ns
Fosfomycin	50	+	+	+	+	+	ns
Gentamicin	10	4 ± 0.05	5 ± 0.12	+	5 ± 0.15	5 ± 0.10	*p* < 0.05
Mezlocillin	75	14 ± 0.05	10 ± 0.15	13 ± 0.20	8 ± 0.20	11 ± 0.10	*p* < 0.001
Oxacillin	1	+	+	4 ± 0.03	+	+	*p* < 0.05
Penicillin	10	13 ± 0.10	9 ± 0.20	14 ± 0.20	9 ± 0.05	11 ± 0.09	*p* < 0.001
Rinfampicin	30	13 ± 0.02	11 ± 0.09	8 ± 0.10	11 ± 0.08	10 ± 0.08	*p* < 0.01
Streptomycin	25	4 ± 0.12	5 ± 0.08	+	4 ± 0.06	4 ± 0.05	*p* < 0.01
Tetracycline	30	14 ± 0.15	10 ± 0.30	7 ± 0.05	8 ± 0.06	7 ± 0.10	*p* < 0.001
Tobramycin	10	4 ± 0.08	4 ± 0.05	4 ± 0.09	3 ± 0.05	4 ± 0.05	ns
Vancomycin	30	+	+	+	+	+	ns

**Table 3:** Antagonistic activity in vitro. The diameter of inhibition halos was recorded in mm and the data were expressed as average ± SD.

Pathogen	LGG	*L. mesenteroides* C1	*L. mesenteroides* C2	*L. mesenteroides* C7	*W. soli* T4	*p*-Value
*S. aureus*	40 ± 0.08	38 ± 0.4	35 ± 0.5	35 ± 0.1	35 ± 0.5	*p* < 0.01
*L. monocytogenes*	30 ± 0.2	29 ± 0.2	30 ± 0.8	31 ± 0.6	30 ± 0.5	ns
*P. aeruginosa*	40 ± 0.07	38 ± 0.5	33 ± 0.5	40 ± 0.1	35 ± 0.6	*p* < 0.01
*S. enterica*	30 ± 0.3	31 ± 0.3	30 ± 0.5	28 ± 0.5	30 ± 0.08	ns

**Table 4:** In vivo resistance to pathogens. Survival assay of N2 worms fed co-cultures of LAB and *P. aeruginosa* or *S. aureus*. The lifespan of worms fed pathogens alone was reported as the control. Three experiments were performed in triplicate for each condition.

*C. elegans* Strain	Diet	Median Lifespan	Maximum Lifespan	Statistics
Wild-type N2	*P. aeruginosa*	3 ± 0.8	5 ± 0.8	-
	LGG + *P. aeruginosa*	5 ± 0.9	9 ± 0.4	*p* < 0.001
	*L. mesenteroides* C2 + *P. aeruginosa*	6 ± 1.2	12 ± 0.8	*p* < 0.001
	*L. mesenteroides* C7 + *P. aeruginosa*	6 ± 0.2	11 ± 0.3	*p* < 0.001
	*S. aureus*	5 ± 0.4	8 ± 0.9	-
	LGG + *S. aureus*	8 ± 0.5	13 ± 0.5	*p* < 0.001
	*L. mesenteroides* C2 + *S. aureus*	7 ± 0.6	11 ± 0.5	*p* < 0.001
	*L. mesenteroides* C7 + *S. aureus*	7 ± 0.9	11 ± 1.2	*p* < 0.001

## Data Availability

Not applicable.
